# Rising HIV infections and shifting transmission patterns in Bangladesh: Emerging risks among key populations

**DOI:** 10.1016/j.pmedr.2026.103423

**Published:** 2026-02-20

**Authors:** Afrin Hawk, Mahmuda Akter, Uthman Okikiola Adebayo, Safayet Jamil

**Affiliations:** a Department of Public Health, Daffodil International University, Dhaka 1216, Bangladesh; b Department of Medical Laboratory Science, Achievers University, Owo, Ondo State, Nigeria; c Department of Medical Laboratory Services, Federal University of Health Sciences Teaching Hospital, Ila Orangun, Osun State, Nigeria; d Department of Public and Community Health, Faculty of Medicine and Health Sciences, Frontier University Garowe, Puntland, Somalia

Dear Editor,

Bangladesh has historically maintained a very low HIV prevalence consistently under 0.1% in the general population and below 1% in most key populations, yet recent national surveillance report points to a worrisome increase in new infections and AIDS-related mortality (Paul, 2020). Annual diagnoses have more than doubled over the past decade, reflecting a consistent upward trend. Although prevalence in the general population remains low, the epidemic is concentrated among key populations, including people who inject drugs (PWID), men who have sex with men (MSM), migrant workers, sex workers, and transgender individuals (Huq et al., 2024). The growing diversity of HIV-1 subtypes, introduced largely through returning migrants, further complicates transmission dynamics and the public health response (Sarker and Jahan, 2023). In this correspondence, ‘key populations’ also refers to adolescents and young adults aged 15–24 years, a group increasingly recognized as vulnerable due to early sexual debut, limited HIV testing uptake, and heightened exposure to stigma.

Shifts in transmission patterns across South Asia confirm the rising importance of sexual transmission, particularly among MSM, compared with earlier epidemics that were driven by PWID (Oyomopito et al., 2015). High HIV incidence among young MSM has been documented in Thailand and Malaysia, identifying this group as a major driver of new infections (Thienkrua et al., 2018). Bangladesh reflects this regional trend: a recent outbreak in Jessore revealed a cluster of HIV-positive students who are MSM, and recent national surveillance estimates now show that MSM represent a substantial share of new infections (Morshed Hemel et al., 2024). This suggests concentrated transmission in educational and youth social networks and underscores gaps in early detection, stigma-sensitive outreach, and HIV prevention programs for young key populations (Yamanis et al., 2024).

Although national epidemic modelling projections suggest that annual new infections may remain modest, such figures obscure the reality of concentrated epidemics within high-risk and marginalized groups, including MSM, PWID, sex workers, and migrant laborers (Korenromp et al., 2024). Regional surveillance studies and multi-source analysis show an increasing concentration of new HIV infections among MSM and transgender women, with structural barriers such as stigma, discrimination, and limited access to testing and treatment sustaining higher transmission rates and undermining public health responses (Korenromp et al., 2024). Although Bangladesh has maintained a long-standing, government-led HIV surveillance system since the late 1990s, important challenges remain in adequately capturing infections among hidden, stigmatized, and younger key populations, particularly MSM (Department of Biochemistry and Molecular Biology, University of Dhaka, Dhaka, Bangladesh., Y K, 2019). Despite an established surveillance system, HIV testing and outreach among key populations especially MSM and young people remain insufficient for timely diagnosis and effective epidemic control. HIV knowledge and prevention practices are particularly poor among vulnerable groups such as urban slum dwellers and younger individuals, who show low awareness and weak engagement in preventive behaviors (Siddique et al., 2025). These limitations suggest that reported figures underestimate the true burden, echoing the World Health Organization and the Joint United Nations Programme on HIV/AIDS concerns for other low-prevalence settings.

Bangladesh is therefore at a critical juncture. The combination of rising incidence, changing transmission dynamics, and emerging outbreaks among key populations signals the risk of a broader epidemic. Strengthening surveillance, expanding voluntary testing and treatment, investing in school- and community-based sex education, and integrating stigma reduction into HIV services must be national priorities (Huq et al., 2024). Early and coordinated action is essential to prevent escalation and to safeguard Bangladesh's commitments to health equity and human rights.

## CRediT authorship contribution statement

**Afrin Hawk:** Writing – original draft, Conceptualization. **Mahmuda Akter:** Writing – original draft, Data curation, Conceptualization. **Uthman Okikiola Adebayo:** Writing – review & editing. **Safayet Jamil:** Supervision.

## Ethical approval

Not applicable.

## Funding

Authors received no external funding.

## Declaration of competing interest

The authors declare that they have no known competing financial interests or personal relationships that could have appeared to influence the work reported in this paper.

## Data Availability

No data was used for the research described in the article.
